# Editorial: Recent Advances in Computer Simulation for Diabetes Treatment and Care

**DOI:** 10.3389/fendo.2022.914657

**Published:** 2022-05-23

**Authors:** Patricio Colmegna, Chiara Toffanin, Marzia Cescon, Roberto Visentin

**Affiliations:** ^1^ Center for Diabetes Technology, University of Virginia, Charlottesville, VA, United States; ^2^ Department of Electrical, Computer and Biomedical Engineering, University of Pavia, Pavia, Italy; ^3^ Department of Mechanical Engineering, University of Houston, Houston, TX, United States; ^4^ Department of Information Engineering, University of Padova, Padova, Italy

**Keywords:** glycemic control, automated insulin delivery, mathematical modeling, *in-silico* trial, glucose sensing, physical exercise, gestational diabetes

The use of computer simulations in diabetes research has permitted to substantially shorten the time between design on a whiteboard and human testing from years to mere months, allowing to test (and possibly discard) different therapies in numerous conditions that would be too complex, dangerous, or unfeasible to perform *in vivo* (1). In this context, this Research Topic compiles six exemplary cases where computer simulation is instrumental in conceptualizing, designing, and evaluating novel approaches for type 1 diabetes (T1D) treatment and care.

A good understanding of what could be the best deployment strategy is a must when designing any new therapy for treating diabetes that is intended to be tested clinically. This topic is covered in the first contribution, where Goez-Mora et al. evaluate performance of an automated insulin delivery (AID) system based on model predictive control (MPC) as they varied the complexity of its formulation, optimization software, and target embedded platform. A Hardware-in-the-loop (HIL) setup was used to conduct this analysis where the portable device that contained the MPC problem and its Kalman filter was connected *via* serial communication to a computer that simulated the model of glucose metabolism. Several performance factors were considered to compare setups, including execution time, accuracy, processor temperature, energy consumption, and glucose control metrics. Based on HIL simulations under time-varying conditions, Quadprog and Raspberry Pi 3 were identified as the best optimization package and embedded system, respectively, to deploy the proposed AID system.

A still open problem in diabetes management is how to control glucose safely during exercise. Despite its clear benefits on weight control, mental health, and sleep, exercise remains a hurdle for most people with T1D due to the increased risk for hypoglycemia. In this regard, the second contribution by Deichmann et al. provides an exhaustive simulation-driven analysis on how current guidelines can protect against exercise-induced acute hypoglycemia if followed correctly. This work also discusses fundamental limitations due to complex interactions between meal and exercise effects on glucose that require further post-exercise adjustments, and the need for models that allow capturing broader scenarios. Despite inherent limitations associated with *in silico* tests, this work confirms that there is still a long road ahead and that more studies are needed to better understand the intricate interplay underlying blood glucose regulation during exercise in T1D.

Despite AID technology is promising, most people with T1D still are under multiple daily injection (MDI) therapy, which needs to be individually tuned for achieving optimal glucose control. This topic is tackled by El Fathi et al., who present a novel methodology for long acting insulin titration under MDI that relies on continuous glucose monitoring (CGM) and smart insulin pen data to finely cover the background insulin needs. Authors show that the proposed algorithm withstands rigorous *in silico* testing using the latest version of the UVA/Padova T1D Simulator. It also outperforms two previous titration strategies based on self-measured blood glucose (SMBG) data by providing robust performance hypoglycemia-wise, especially overnight. Since there is always a gap between simulation and reality, this algorithm will be now integrated into a decision support system for its final evaluation in a clinical setting.

The main bottleneck to improve time in target range with a hybrid approach or to safely transition to a fully automated closed-loop solution is the slow absorption and action of current insulin analogs. The reason is that making a controller more aggressive under this condition will inevitably lead to higher risk for late hypoglycemia due to insulin stacking. The fourth contribution, by Villa-Tamayo et al., revolves around this idea by proposing an adaptive insulin on board (IOB) safety layer that can be easily integrated into the control strategy. To account for intrasubject variability, low, mid, and high IOB upper bounds are dynamically generated with an interval model driven by basal-bolus open-loop therapy actions. According to glycemic and plant-model mismatch estimates, one of the upper bounds is selected to allow for more/less aggressive control actions in case imminent risk for hyper/hypoglycemia is detected. A battery of tests was performed with the commercial version of the UVA/Padova T1D Simulator (2), showing the efficacy of the proposed approach in proactively adjusting the controller constraints under intrasubject variability.

In most artificial pancreas schemes, the glucose level is measured by means of a CGM sensor. As alternative, Olçomendy et al. propose an approach where an islet-based biosensor is used instead. This technology offers a more comprehensive representation of the patient’s current metabolic state that can be leveraged by an AID system to better regulate glycemia in T1D. In this contribution, authors test a fully-closed loop biosensor-based controller personalized using genetic-algorithm optimization and a hybrid robust CGM-based controller that covers intersubject model uncertainty, i.e., a one-fits-all solution. Both approaches are evaluated under subject-specific meal scenarios, showing promising results. Authors discuss how lack of personalization could come at the detriment of performance, and propose a strategy where data coming from the CGM and biosensor are integrated into a more general AID framework.

Not all populations with T1D have the same insulin needs. For instance, it is well known that tailored therapy solutions are vital for pregnant women with T1D to appropriately account for significant metabolic changes that occur as pregnancy advances. The final contribution by Ozaslan et al. sheds light on how a Zone-MPC AID system should be adapted to satisfy pregnancy-specific clinical requirements. Through exhaustive analysis, authors select a set of tunable controller parameters, including, among others, the day-time and night-time target glucose zones, and the meal bolus insulin decay curve. These parameters are then tuned to safely tighten glucose control under various challenging conditions that mimic different pregnancy stages. Simulations are performed using the *in silico* adult cohort of the UVA/Padova T1D Simulator, adding extreme hyper- and hypoglycemia conditions to test robustness of the proposed solution. Authors also report preliminary results of a pilot clinical study in 8 pregnant women with T1D over 48 hours to support their *in silico* findings, and validate safety and feasibility of their pregnancy-specific AID solution.

## Author Contributions

The editorial article has been drafted by PC, edited, and reviewed by CT, MC and RV. All the authors listed in the Editorial have made a substantial, direct, and intellectual contribution to the work. All authors contributed to the article and approved the submitted version.

## Conflict of Interest

Dr Cescon serves on the advisory board for Diatech Diabetes.

The remaining authors declare that the research was conducted in the absence of any commercial or financial relationships that could be construed as a potential conflict of interest.

## Publisher’s Note

All claims expressed in this article are solely those of the authors and do not necessarily represent those of their affiliated organizations, or those of the publisher, the editors and the reviewers. Any product that may be evaluated in this article, or claim that may be made by its manufacturer, is not guaranteed or endorsed by the publisher.
